# Reuse of LiCoO_2_ Electrodes Collected from Spent Li‐Ion Batteries after Electrochemical Re‐Lithiation of the Electrode

**DOI:** 10.1002/cssc.202100629

**Published:** 2021-05-06

**Authors:** Katja Lahtinen, Eeva‐Leena Rautama, Hua Jiang, Samuli Räsänen, Tanja Kallio

**Affiliations:** ^1^ Department of Chemistry and Materials Science School of Chemical Engineering Aalto University P.O. Box 16100 00076 Aalto Finland; ^2^ Department of Applied Physics, School of Science Aalto University P.O. Box 15100 0076 Aalto Finland; ^3^ Umicore Finland B.O. Box 286 67101 Kokkola Finland

**Keywords:** Doping, energy conversion, lithiation, lithium-ion batteries, renewable resources

## Abstract

The recycling of used Li‐ion batteries is important as the consumption of batteries is increasing every year. However, the recycling of electrode materials is tedious and energy intensive with current methods, and part of the material is lost in the process. In this study, an alternative recycling method is presented to minimize the number of steps needed in the positive electrode recovery process. The electrochemical performance of aged and re‐lithiated Mg−Ti‐doped LiCoO_2_ and stoichiometric LiCoO_2_ was investigated and compared. The results showed that after re‐lithiation the structure of original LiCoO_2_ was restored, the capacity of an aged LiCoO_2_ reverted close to the capacity of a fresh LiCoO_2_, and the material could thus be recovered. The re‐lithiated Mg−Ti‐doped LiCoO_2_ provided rate capability properties only slightly declined from the rate capability of a fresh material and showed promising cyclability in half‐cells.

## Graphical abstract

**Electrode recycling**: Doped and undoped LiCoO_2_ electrodes collected from aged pouch cells are re‐lithiated electrochemically using metallic lithium as a counter electrode. The performance of an aged and re‐lithiated Mg−Ti‐doped LiCoO_2_‐containing electrode is close to the performance of the fresh material, which is promising from a recycling point of view.

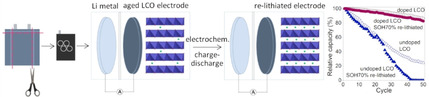

## Introduction

The demand for lithium‐ion battery applications in both consumer electronics and electric vehicles is increasing rapidly. Between 2010 and 2018 the annual increase in demand has been 30 % with the 2018 volume being 180 GWh, and in the future the market is expected to continue growing approximately 25 % annually.^[1]^ This will lead to resource shortages and increases in the prices of several critical metal elements, such as lithium and cobalt, vital for the electrode production.[^1^, ^2^, ^3^] Because of this, recycling and reusing materials in lithium‐ion batteries has become an interesting topic. The first papers were published already in the turn of the century[^4^, ^5^, ^6^] but in the 2010s the research interest has skyrocketed.

Generally, three alternative recycling methods for lithium‐ion batteries exist: hydrometallurgy‐, pyrometallurgy‐ and biometallurgy‐based routes.[^7^, ^8^] Hydrometallurgy‐based routes typically have a high extraction rate, a high metal selectivity and a low energy consumption.[^4^, ^9^, ^10^, ^11^, ^12^, ^13^, ^14^, ^15^] However, the processes are often complicated with several steps and utilize concentrated acids, which easily generates large amounts of waste solutions. Pyrometallurgy‐based methods have a high efficiency and they are easy to scale up.[^16^, ^17^] On the other hand, the high temperatures needed in the processes lead to high energy consumption and emissions. In addition, the recovery of lithium is difficult.[^7^, ^17^] Biometallurgy‐based methods utilize bacteria to extract metals from the spent lithium‐ion batteries.[^18^, ^19^] The methods are usually quite inexpensive, but as a downside the extraction processes are slow.^[20]^

Lithium‐ion batteries can be recycled either by processing the whole battery,[^21^, ^22^, ^23^, ^24^] or dismantling the cells before starting the recovery process.[^5^, ^6^, ^9^, ^12^, ^14^, ^19^, ^25^, ^26^, ^27^, ^28^, ^29^, ^30^, ^31^] The latter has been more popular in the literature, but the dismantling can be a laborious process without proper equipment. One could argue that this method is not easy to scale‐up for industrial purposes. However, for example Nan et al.^[12]^ reported about 5000 spent cells dismantled in 1 h, which indicates that scaling up should be possible. The advantage of recycling a whole lithium‐ion battery is that the possibly challenging dismantling process is skipped, and thus one process step is reduced. However, having all the battery components in the same material flow initially can increase the amount of impurities in the final product. Additional process steps might be necessary to reduce the impurity concentrations in the recycled material.^[24]^ Even if most of the elements are recovered with the above‐mentioned methods, the typical layered structure of the metal oxide intercalation compounds is almost certainly lost, and the material downgraded. Typically, the positive electrode materials are reduced to more low‐value chemicals, such as CoSO_4_,[^4^, ^12^, ^23^, ^24^, ^31^] Co(OH)_2_,[^5^, ^9^, ^32^] CoCO_3_,[^8^, ^22^] and Li_2_CO_3_,[^4^, ^9^, ^12^, ^24^, ^31^, ^32^] during the recycling process. To synthesize these compounds back to Li‐ion battery electrode materials requires energy. Therefore, if the structure of the original electrode material can be spared during the recycling process, potentially both energy and money will be saved. Up to date, studies about LiCoO_2_ reuse without material decomposition are rare, though recently a few have been published.[^33^, ^34^, ^35^] Zhang et al.,^[33]^ for example, have recently shown that the re‐lithiation of Li_*x*_CoO_2_ electrode is possible without removing the active material from the current collector foil. However, in all these studies, LiCoO_2_ was at some point removed from the current collector. Therefore, interestingly, despite the intensive study on lithium‐ion battery recycling, the use of cycled electrodes without removing the active material from them has not been investigated thus far.

With the commercial success of Li‐ion batteries, it is natural that their aging mechanisms have been investigated widely. The processes leading to the capacity fade include electrolyte decomposition, Li deposition on the negative electrode, active material dissolution, structural changes in the active material, separator pore closure and increase in the cell stack pressure.[^36^, ^37^, ^38^, ^39^, ^40^] While all the above‐mentioned mechanisms do affect the cell capacity, the electrolyte decomposition through the formation of a solid electrolyte interphase (SEI) on the negative electrode can be considered as the most dominant. The SEI formation consumes lithium, which leads to the loss of lithium in the positive electrode. While the loss of lithium decreases the capacity of the whole cell, the positive electrode may stay relatively unharmed with only small structural changes observed.^[41]^ If the original structure of the positive electrode can be preserved during the recycling process instead of the above‐mentioned material downgrading, both the energy consumption and the synthesis expenses will be reduced.

In the post‐mortem studies of aged lithium‐ion batteries, electrodes extracted from spent batteries have been used to investigate the aging processes of the materials. Both half cells,[^42^, ^43^, ^44^, ^45^, ^46^, ^47^] with negative or positive electrode as a working electrode and metallic Li as a reference, and reconstructions to full cells[^43^, ^44^, ^48^] have been used. However, to the best of our knowledge, the research has focused solely on the aging mechanisms while no comprehensive electrochemical investigation focusing on the reuse has been conducted. On the other hand, these studies have proved that constructing properly working cells using aged electrodes is possible. Therefore, in this work the recovered positive electrodes from the aged pouch cells are assembled into half cells, and their electrochemical properties are tested keeping the material recycling in mind.

## Results and Discussion

### Characterization of the aged and re‐lithiated electrodes

The LiCoO_2_/graphite pouch cells were aged to two different state of health (SOH) values of 90 and 70 % in the voltage range of 3.0–4.4 V. Typically, capacity loss to SOH 80 % is considered as the end‐of‐the‐life criteria for Li‐ion batteries. These investigated pouch cells have earlier been shown to provide long cycle life in the voltage range of 3.0–4.2 V.^[49]^ For this study, the relatively high upper voltage limit of 4.4 V was chosen to provide information in an industrially relevant voltage range, and the relative capacity loss of the investigated materials during the cycling is presented in Figure S1 in the Supporting information. After reaching the targeted SOH, the aged LiCoO_2_ (LCO) electrodes from the fully discharged batteries (i. e., with LCO lithiated) were collected and electrochemically re‐lithiated. Two different LCOs were used: essentially stoichiometric LCO with the experimental composition of Li_0.97_CoO_2_ (abbreviated as S−LCO) to represent a standard LiCoO_2_, and Mg−Ti doped LCO with the stoichiometry of Li_0.97_(Mg_0.005_Ti_0.002_Co_0.994_)O_2_ (abbreviated as D−LCO).

The chemical composition, structure, and morphology of the original, aged, and electrochemically re‐lithiated LCO electrodes were investigated using X‐ray diffraction (XRD), Raman spectroscopy, electron energy loss spectroscopy (EELS) and scanning electron microscopy (SEM). The compositions of the original LCOs provided by the material manufacturer are presented in Table S1 in the Supporting information. The Li/Co ratio is observed to be slightly below 1 for both the investigated materials, most likely due to Li loss during synthesis.^[49]^ D−LCO is observed to contain 0.5 mol% of Mg and 0.2 mol% of Ti.

**Figure 1:**
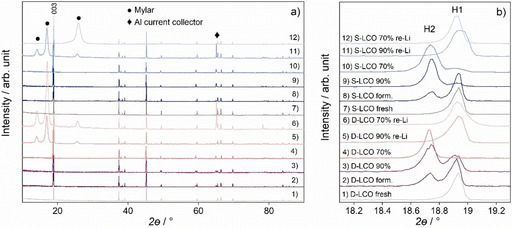
(a) XRD patterns of the formatted, aged, and re‐lithiated S−LCOs and D−LCOs. (b) Magnification of the data near the divided (003) reflection at 18.6–19.0°.

**Figure 2:**
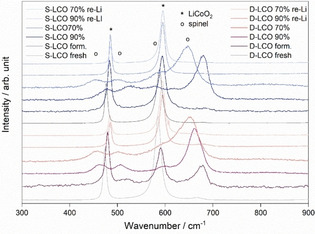
Raman spectra of the formatted, aged, and re‐lithiated LCO samples.

XRD and Raman spectroscopy offer information about the structure of the aged and re‐lithiated samples, and the patterns of the investigated materials are presented in Figures 1 and 2. Based on the XRD data, both the LCO active materials have well‐defined, crystalline structure. The compositions of the mixed materials were analyzed using the Le Bail method and the program Fullprof.^[50]^ Two separated phases are observed in both the formatted and the aged materials. The first of the phases corresponds to the typical rhombohedral structure of lithium cobalt oxide (analyzed using a hexagonal setting and noted here as H1). The second phase corresponds best to a similar structure (noted as H2) but with an expanded lattice, 2*a*_H1_×2*a*_H1_×≈*c*_H1_, resulting from vacancy ordering and adapted from the work by Yahia et al.^[51]^ The vacancy‐ordered cell was chosen as the fitting results at certain areas were better against a non‐ordered cell, although the actual supercell reflections could not be evidenced with the laboratory XRD. Representative samples were air‐protected by a Mylar film during measurements (reflections marked with a dot), and occasionally the Al current collector is visible as well (marked with a diamond). Other crystalline impurities are not observed within the limits of a powder XRD. The reliability factor values for the weighted profile in the Rietveld analysis (*R*_wp_) are between 11–19 % while the expected reliability factor of the data in the Rietveld analysis (*R*_e_) for present data is 7–10 % due to the relatively small amount of the sample available.

Lattice parameters are presented in Table 1. The values show that for fresh S−LCO and D−LCO, the lattice parameter *a* is approximately the same while the lattice parameter *c* is smaller for S−LCO than for D−LCO. For the formatted samples, the *a* parameter is slightly larger and the *c* parameter smaller for S−LCO compared to D−LCO in both the observed phases. A plausible explanation for the difference is the size of the dopants,[^49^, ^52^, ^53^, ^54^] and the pristine structure of the materials is presented in more detail in our previous work.^[49]^ During the aging, changes occur in the lattices of the materials. The *c* parameters of both the phases increase for the stoichiometric and doped material except for the S−LCO sample aged to 90 %. The increase in *c* parameter of LiCoO_2_ is attributed to decreasing state of charge (SOC),^[55]^ in other words with the loss of Li, and this is assumed to occur in the aged materials as well. This finding is supported by the analysis of the negative electrode and will be discussed later. The *a* parameter varies between the samples, with the value decreasing for S−LCO upon aging but slightly increasing for D−LCO. Based on the earlier literature, the *a* parameter is expected to decrease upon aging due to the increase of the average valence state of Co upon Li extraction. However, the reversed behavior of D−LCO is contradictory to this. One possible explanation is the gradual decrease of the average Co valence during the aging of the doped sample, which could be manifested through the formation of oxygen vacancies in the CoO_2_ layers. However, typically vacancies are found when the loss of Li in Li_*x*_CoO_2_ exceeds *x*≈0.5.[^56^, ^57^]

**Table 1:** Selected structural parameters of the formatted, aged, and re‐lithiated LCO samples extracted from the LeBail fittings. Phase fractions (H1/H2) are visual estimates based on the structure simulations.^[a]^

Sample	*a*_H1_ [Å]	*c*_H1_ [Å]	*a*_H2_ [Å]	*c*_H2_ [Å]	H1/H2	(*I*_012_+*I*_006_)/*I*_101_
S−LCO fresh	2.8162	14.053	–	–	100:0	0.57
S−LCO form.	2.8158	14.055	5.6258	14.194	80 : 20	–
S−LCO 90 %	2.8145	14.062	5.6251	14.191	10 : 90	–
S−LCO 70 %	2.8147	14.064	5.6245	14.199	10 : 90	–
S−LCO 90 % re‐Li	2.8164	14.054	–	–	100:0	–
S−LCO 70 % re‐Li	2.8161	14.053	–	–	100:0	0.77
D−LCO fresh	2.8161	14.062	–	–	100:0	0.53
D−LCO form.	2.8148	14.057	5.6231	14.194	70 : 30	–
D−LCO 90 %	2.8156	14.068	5.6247	14.199	35 : 65	–
D−LCO 70 %	–	–	5.6236	14.225	0 : 100	–
D−LCO 90 % re‐Li	2.8161	14.058	–	–	100:0	–
D−LCO 70 % re‐Li	2.8160	14.058	–	–	100:0	0.64

[a] Phase 1: *R*‐3 *m* (#166), hexag. setting *a*_H1_×*a*_H1_×*c*_H1_. Phase 2: *R*‐3 *m* (#166), hexag. setting 2*a*_H1_×2*a*_H1_×≈*c*_H1_.

Both the *a* parameter and the *c* parameter of the re‐lithiated samples have similar values to the fresh samples, indicating that the stretch caused by the removal of Li is reverted. The samples re‐lithiated after longer cycling the (SOH 70 %) have similar lattice parameters compared to the samples after shorter cycling discontinued to SOH 90 %. This indicates that at least within the SOH of 70–100 %, the LCO materials can be revived similarly, which is also supported by the appearance of the phases H1 and H2. For both the materials the amount of H2 increases with the aging while the amount of H1 decreases. Based on the length of the *c* parameter, the H2 phase corresponds to approximately *x*=0.75 in Li_*x*_CoO_2_ ^[58]^ and hence, its appearance indicates that Li is lost from the material with aging. It should be noted that even in the formatted samples, H2 is observed designating that a considerable amount of Li is lost already during the first cycle of the aging. In the re‐lithiated samples only H1 is observed denoting that the lost lithium is restored in the lattice.

To understand the crystal structures of the re‐lithiated materials better, the intensity ratio of the Bragg reflections (*I*_012_+*I*_006_)/*I*_101_ of the samples after the aging to SOH 70 % were calculated and compared to the fresh materials. The intensity ratio is related to the level of hexagonal stacking order,^[59]^ increasing value indicating decreased ordering. The calculated values are collected in Table 1 and they suggest that the stacking order decreases in both the materials after re‐lithiation. Furthermore, S−LCO experiences more significant decrease after re‐lithiation compared to D−LCO. This decline is explained by the structural changes occurring during the aging to SOH 70 % and the following re‐lithiation process as indicated by the XRD patterns in Figure 1.

The Raman spectroscopy results support the observations done from the XRD diffractograms. Figure 2 shows that the characteristic modes of *e_g_* and *a_g1_* for hexagonal LiCoO_2_ at 485 and 595 cm^−1^, respectively, are observed in the fresh and the formatted samples of S−LCO and D−LCO. The *e_g_* band is caused by O−Co−O bending vibrations and the *a_g1_* by Co−O stretching.[^60^, ^61^] However, when the materials are aged to SOH 90 %, the characteristic bands corresponding to a cubic spinell (*Fd‐*3 *m)* phase at 460, 506, 602 and 670 cm^−1^ become more prominent than the LiCoO_2_ bands, and at SOH 70 % only the spinel bands are visible. These spinel modes are observed in the formatted D−LCO sample as well and correspond to *e_g_*, *f* ^*1*^_*2g*_, *f* ^*2*^_*2g*_ and *a_g_*, respectively. The observed spinel bands are typical for both the Co_3_O_4_ and *Fd‐*3 *m* phase of Li_*x*_CoO_2_ (*x*=0.5), and therefore it cannot be definitely determined which of the materials is present. The formation of the spinel has been reported in studies investigating the aging of LiCoO_2_,[^62^, ^63^] and therefore its appearance in the SOH 90 % and SOH 70 % samples is expected. The disappearance of the *e_g_* and *a_g1_* bands of LiCoO_2_ is explained by their behavior upon cycling. Raman studies investigating the lithium intercalation/deintercalation in LiCoO_2_ have shown that the intensity of the *e_g_* and *a_g1_* bands decreases notably when Li is extracted from the structure. This is due to the increasing conductivity of the material which results in the reduced optical skin depth of the laser beam.[^60^, ^64^, ^65^, ^66^] The laser beam has been reported to have the penetration depth of around 50–100 nm using a 514.5 nm green laser beam.^[67]^ Therefore, it can be concluded that the particles are measured only relatively close to surface (particle diameter is 16 μm as shown below by SEM) where the structural changes can be assumed to be stronger. Combined with the decreasing intensity of the LiCoO_2_ bands, mostly spinel bands are observed in the data even if most of the sample still is delithiated LiCoO_2_. Indeed, the XRD results show that while there are changes in the lattice, LiCoO_2_ does not decompose upon aging. Similar behaviour has also been observed in previous studies.[^68^, ^69^] The appearance of the spinel bands in the formatted D−LCO is explained by the smaller primary particles observed in our previous study:^[49]^ The smaller particles lead to higher surface area and thus the larger appearance of the lithium‐vacant structures formed during the formation.

The peaks are also observed to move to smaller wavenumber upon aging, which is related to changes in the chemical bond length of the material with a smaller wavenumber indicating a longer bond. Therefore, the shift upon aging indicates the lengthening of the chemical bonds within the investigated LCOs. This is in agreement with the observed increase in the lattice parameter *c* in the XRD results, and it further confirms the Li loss in the aged materials. The re‐lithiated samples show similar results to the fresh ones with two clear bands of hexagonal LiCoO_2_ at 485 and 595 cm^−1^, and only a very small band at 670 cm^−1^. As a low amount of the spinel phase is observed in the fresh materials as well, the 670 cm^−1^ band can be concluded not to be formed during the aging of the electrodes. This supported by XRD indicates that the original hexagonal structure of LiCoO_2_ is restored upon the re‐lithiation.

**Figure 3:**
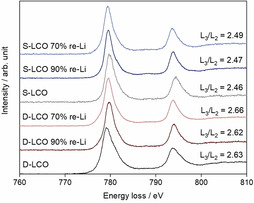
EELS spectra of Cobalt L‐edge for the pristine and re‐lithiated samples.

To further elucidate the structure of the reverted active materials, the energy loss spectra of cobalt L edges for the re‐lithiated materials acquired in the EELS measurements are presented in Figure 3. The L edge is sensitive to the valence state of transition metals.[^70^, ^71^, ^72^, ^73^] The L_3_ edge is induced by the electron transitions from 2p_3/2_ orbital to unoccupied 3d_3/2_3d_5/2_ orbital and the L_2_ edge by the transition from 2p_1/2_ orbital to 3d_3/2_ orbital. There is only a little variation observed in the L_3_ edge position between the samples, the energy loss being 779.6–780.0 eV. Similarly, the L_2_ edge position varies slightly between 793.7–794.1 eV. Hence, the results suggest that there are no substantial differences between the samples in the valence state of cobalt.

The method by Wang et al.^[71]^ is used for the L_3_/L_2_‐edge intensity ratio calculations from the EELS measurements. This ratio is related to the unoccupied states of the 3d orbital and provides information about the valence states.[^70^, ^71^] The higher intensity ratio indicates a lower valence state and an obvious difference is observed between S−LCO and D−LCO. The L_3_/L_2_ intensity ratios for S−LCO are 2.46–2.49 depending on the sample while they are 2.62–2.66 for the D−LCO samples. Although these values are within a range typically observed for cobalt in LiCoO_2_,^[71]^ they also indicate that the valence state of D−LCO is slightly lower than that of S−LCO. A similar observation has been done in our previous work.^[49]^ The lowered valence state of cobalt enhances the conductivity of the material while its presence is attributed to the Mg−Ti doping. It is also observed that there is no difference between the fresh materials and the re‐lithiated samples, which further supports the observations that the materials can be recovered to their original forms with the electrochemical re‐lithiation.

**Figure 4:**
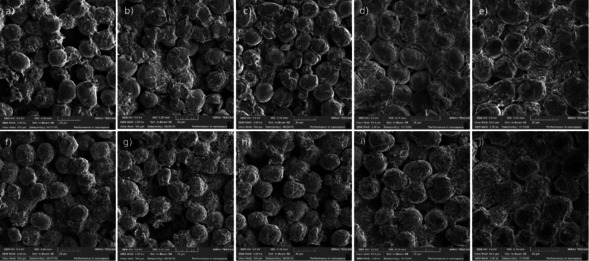
SEM images of S−LCO (a) after formation, (b) at SOH 90 %, (c) at SOH 70 %; (d) re‐lithiated SOH 90 %, (e) re‐lithiated SOH 70 %; D−LCO (f) after formation, (g) at SOH 90 %, (h) at SOH 70 %; (i) re‐lithiated SOH 90 % and (j) re‐lithiated SOH 70 %. Scale bars: 20 μm

The SEM images of the investigated electrodes are presented in Figure 4 to visualize changes in the morphology induced by the aging and re‐lithiation. Both S−LCO and D−LCO have the mean secondary particle size of approximately 16 μm as seen in the images taken after the cell formatting (Figure 4a,d). However, D−LCO seems to have prominent primary structure, while in S−LCO a secondary particle consists of only a few primary particles. After cycling S−LCO to SOH 90 %, a few cracks can be observed (Figure 4b), and similar behavior is visible at SOH 70 % (Figure 4c) as well. However, it should be noted that most of the particles look relatively unchanged. The cracking of the particles is not observed for the D−LCO particles during the cycling (Figure 4e,f). However, small slits between the particles and the surrounding binder are observed, which indicates that volume changes during the delithiation/lithiation process have taken place in the material. In the re‐lithiated materials, no additional cracks are observed while the primary structure observed in the formatted and aged samples is also still well visible. The results further verify that the LCO particles retain most of their initial properties after the electrochemical re‐lithiation.

While the focus of this study is on the positive electrode, some attention should be paid to the negative graphite electrode of the pouch cells. To investigate the loss of lithium, atomic absorption spectrometry (AAS) was used to measure the amount of Li in the graphite electrodes and the results are summarized in Table S2 in the Supporting Information. The amount of lithium in the graphite electrodes is observed to increase with the pouch cell aging indicating that lithium is indeed lost to graphite during the aging. It should also be noticed that the amount of lithium lost in S−LCO is larger than the amount lost in D−LCO suggesting that the thickening of the SEI layer is faster in the pouch cells with the former positive electrodes. The phosphor concentration in the graphite electrodes is found out to increase upon the pouch cell aging. This supports the observations about the thickening SEI layer, as it indicates the decomposition of the phosphor containing electrolyte salt.

### Reuse of the re‐lithiated LiCoO_2_s

**Figure 5:**
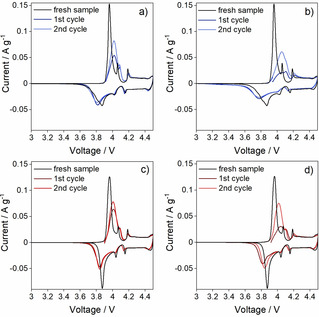
CV plots of (a) S−LCO at SOH 90 %, (b) S−LCO at SOH 70 %, (c) D−LCO at SOH 90 % and (d) D−LCO at SOH 70 % in the voltage range of 3.0–4.5 V compared to the CV plots of the fresh samples.

To investigate the electrochemical properties of the aged LCO materials, positive electrodes were extracted from the cycled full cells and reassembled in half cells. In the voltammograms, the first cyclic voltammetry (CV) cycle represents the amount of lithium in the aged electrode and the second CV cycle the result of the re‐lithiation in the aged electrode. Three peaks during both the Li insertion and extraction scans are observed for S−LCO and D−LCO (Figure 5). The largest peak at 3.96/3.87 V is caused by the lithium deintercalation/intercalation reaction in the two‐phase domain. The two smaller peaks at 4.06 and 4.20 V originate from Li‐ion ordering within the LiCoO_2_ structure, the symmetry turning from rhombohedral to monoclinic during the first peak and then back to rhombohedral during the second peak. The peaks are sharper for the fresh samples compared to the electrodes extracted from the cycled cells, especially S−LCO at SOH 70 %. There are two possible explanations for this. First, the peak broadening could indicate that the resistances of the electrodes increase during the cycling. Secondly, the fresh samples have not been collected from pouch cells but prepared for this measurement. While the compositions of the electrodes are the same, the used conductive carbon is different and the active material loading is lower in the fresh sample (8.0 mg cm^−2^ for the fresh, 11.0–14.5 mg cm^−2^ for the aged sample), which could lead to differences in the cell resistance.

It can also be observed in Figure 5 that the first scan of the electrodes extracted from the full cells shows considerably lower lithium extraction peak than the second cycle. As the integrated area of the current‐voltage plot corresponds to the capacity of the investigated material, it is deducted that the capacity increases between the first and the second cycle when the aged materials are cycled in the half cells. The calculated capacities for the aged materials’ first Li extraction cycle are 148, 104, 160 and 120 mAh g^−1^ for S−LCO SOH 90 % and 70 % and D−LCO SOH 90 % and 70 %, respectively. The recovered capacities are approximately the same for S−LCO and D−LCO, around 180 mAh g^−1^, and only slightly smaller than the corresponding fresh material capacities (184 mAh g^−1^). The capacity loss of the LCO electrodes cycled in high voltages in full cells is usually attributed to irreversible phase transformations,[^74^, ^75^] Co dissolution,[^76^, ^77^] particle cracking[^75^, ^78^] or the loss of lithium due to SEI layer formation on the negative electrode.[^36^, ^79^, ^80^] However, as the second scan provides a taller peak and thus a higher capacity, some of the lost capacity is clearly recovered during the first Li insertion (i. e., re‐lithiation cycle). In other words, with a fresh metallic lithium counter electrode, the LCO materials are electrochemically re‐lithiated. In addition, it is observed that the capacity loss is expectedly more severe with extended cycling in the full cells. This indicates that one form of capacity loss in the pouch cells is the Li loss, which is supported by the AAS results showing increase in the Li content in the graphite electrode upon aging. Based on these results, the reusability of the re‐lithiated materials is investigated.

**Figure 6:**
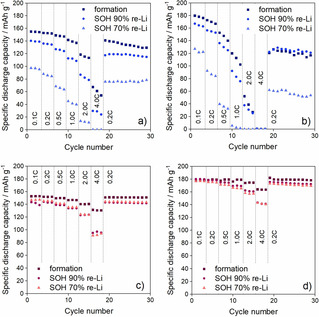
Rate capabilites of the LCO electrodes recovered from the aged pouch cells and re‐lithiated. (a) S−LCO in the voltage range of 3.0–4.3 V, (b) S−LCO in the voltage range of 3.0–4.5 V, (c) D−LCO in the voltage range of 3.0–4.3 V and (d) S−LCO in the voltage range of 3.0–.5 V.

To investigate the reusability of the LCO materials after the re‐lithiation of the aged electrodes, rate capability measurements were performed in two different voltage ranges, 3.0–4.3 and 3.0–4.5 V. The voltage ranges were selected to match the typical range of LCO (3.0–4.3 V) and the commercially attractive range of positive electrodes (3.0–4.5 V). The results shown in Figure 6 indicate that the specific discharge capacity of the formatted sample is sligtly above 150 mAh g^−1^ with 0.1 C current in the voltage range of 3.0–4.3 V, and approximately 180 mAh g^−1^ in the voltage range of 3.0–4.5 V for both S−LCO and D−LCO. When the effect of the aging on the recovery of S−LCO and D−LCO is compared, it is seen that the initial capacity of the aged and re‐lithiated electrodes is higher for D−LCO than it is for S−LCO. For example, in the voltage range of 3.0–4.3 V, after aging the pouch cells to SOH 70 %, the specific discharge capacities are 98 and 147 mAh g^−1^ for re‐lithiated S−LCO and D−LCO, respectively. In other words, D−LCO is able to recover from the aging better than S−LCO, and therefore, it provides higher specific capacity. Similar results are seen in the voltage range of 3.0–4.5 V as well, the specific discharge capacities of the re‐lithiated materials collected from the pouch cells aged to SOH 70 % being 127 and 177 mAh g^−1^ for S−LCO and D−LCO, respectively (0.1 C current). These results differ slightly from the CV results presented in Figure 5 as the CV suggested similar capacity recovery for both S−LCO and D−LCO during the re‐lithiation. However, the second cycles of the CVs have been measured with the low scan rate of 0.02 mV s^−1^ corresponding to the C‐rate of app. 0.05 C. The higher C‐rate at the rate capability measurements can partially explain the difference. In addition, the capacity retention of the aged S−LCO after the re‐lithiation can be observed to be very poor in Figure 6b suggesting the poor cyclability of this recovered material. Here, the difference in the recovered capacities indicates irreversible capacity loss in the material, most likely caused by the degradation of the electrode. The XRD results (Table 1) show that after the re‐lithiation S−LCO has poorer stacking order compared to D−LCO, and this could explain the differences in the recovered capacities. Moreover, the degradation might also have occurred in the electrode additives instead of the active materials, which is discussed more in the electrochemical impedance spectroscopy (EIS) section.

There is a difference between the rate capability properties of the re‐lithiated S−LCO and D−LCO as well. The specific discharge capacities with 4 C current are 61 and 0 mAh g^−1^ for formatted S−LCO in the voltage ranges of 3.0–4.3 and 3.0–4.5 V, respectively, while they are 131 and 163 mAh g^−1^ for formatted D−LCO in the same voltage ranges. Based on Figure 6, the decrease in the rate capability properties depends on the material and voltage range. For S−LCO aged to SOH 70 %, the specific discharge current with 4 C is 0 mAh g^−1^ in the voltage range of 3.0–4.3 V. The drop from 0.1 to 0.2 C is slightly larger than it is for the formatted sample. However, the decline could also be caused by the poor cyclability of the material in high C‐rates, which is seen by comparing the parallel discharge capacities to each other. The performance of S−LCO after the aging and re‐lithiation is even poorer in the voltage range of 3.0–4.5 V, the capacity drop between 0.1 and 0.2 C cycles being more than 30 mAh g^−1^ and the specific discharge capacity dropping to 0 mAh g^−1^ already at 1 C. The rate capability properties of D−LCO change less during the aging. The specific discharge capacities at 4 C for the re‐lithiated D−LCOs aged to SOH 70 % are 92 and 142 mAh g^−1^ in the voltage ranges of 3.0–4.3 and 3.0–4.5 V, respectively. The capacity decrease within cycles with the same C‐rates is also very small, which indicates good stability.

**Figure 7:**
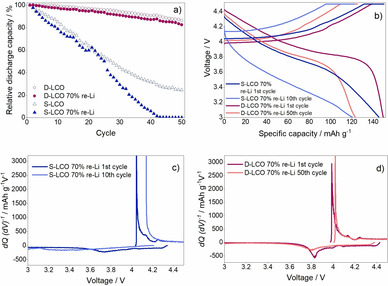
Re‐cycling of the re‐lithiated LCOs aged to SOH 70 % compared to the fresh materials. (a) Relative discharge capacity decrease of the re‐lithiated compared to the fresh materials; (b) charge–discharge curves; differential capacity of (c) S−LCO and (d) D−LCO.

At the end of the rate capability test the half cells were cycled again with 0.2 C to investigate the capacity retention of the re‐lithiated LCOs. D−LCO showed stable performance while the specific capacity of S−LCO varied more. To investigate the cyclability of the aged and re‐lithiated LCOs more, they were cycled in half cells using the voltage range of 3.0–4.5 V and the C‐rate of 0.5 C. The stability of the re‐lithiated stoichiometric and the Mg−Ti doped LCOs aged to SOH 70 % is presented in Figure 7. It is clearly seen that while the initial capacities of the materials are similar, the capacity retention of D−LCO is good compared to S−LCO. After 50 cycles, the specific capacity of the re‐lithiated S−LCO is 0 mAh g^−1^ while it is 125 mAh g^−1^ for D−LCO. In Figure 7a the relative discharge capacity retention of the re‐lithiated materials is compared to the fresh ones. The cyclability of the re‐lithiated materials are observed to be only slightly poorer than those of the fresh materials. After 50 cycles, the capacity retention of re‐lithiated D−LCO is 83 % while it is 86 % for fresh D−LCO. Considering the high cut‐off voltage of the cycling, this result is very promising from the recycling perspective.

To investigate the processes behind the differences in the cycle lives, differential capacity was plotted against voltage, as is seen in Figure 7c,d. The 10th cycle has been selected for S−LCO and the 50th cycle for D−LCO as the SOH of these re‐lithiated materials are approximately the same at these cycles numbers. The main lithiation/delithiation peak is observed to move to higher voltage upon cycling, which indicates that the polarization of the electrode increases with the cycle number. It should be noted that while the decrease is small for D−LCO (0.03 V) during 50 cycles, the increase for S−LCO is bigger (0.10 V) only after 10 cycles.

**Figure 8:**
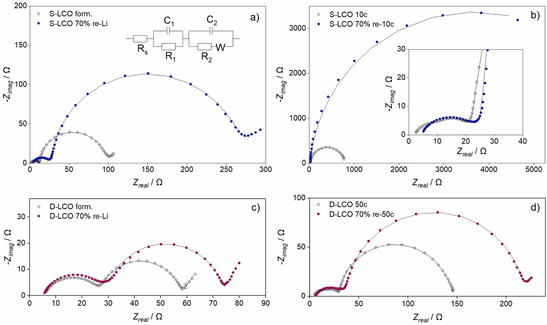
Evolution of the complex‐plane diagrams of the aged LCOs in three electrode cells at the SOC of 50 % cycled in the voltage range of 3.0–4.5 V. (a) Stoichiometric LCO after re‐lithiation, (b) stoichiometric LCO 10 cycles after the re‐lithiation, (c) Mg−Ti doped LCO after the re‐lithiation, (d) Mg−Ti doped LCO 50 cycles after the re‐lithiation. Experimental data is presented as dots and fitted data based on the equivalent circuit as lines.

To further understand the changes in internal resistances induced by the cycling of the re‐lithiated electrodes, the impedance spectra were measured, and the data is presented in Figure 8. The complex‐plane plots of the three electrode cells consist of two semicircles at high and middle frequencies and a straight line at low frequencies. The semicircle at high frequency is attributed to the interface between the active material and the current collector.[^81^, ^82^] The larger semicircle at mid‐frequencies corresponds to the charge‐transfer resistance in the investigated electrode. The line at low frequencies originates from solid‐phase diffusion.

The kinetic parameters based on the equivalent circuit presented in Figure 8a are collected in Table 2. The equivalent series resistance (*R*_S_) is observed to stay relative similar in all measurements although the S−LCO values are slightly smaller than the others. The small differences are most likely caused by small variations in the cell set‐up and minorF errors in data fitting. The active material/current collector interphase resistance (the high‐frequency semicircle) is observed to be larger for the re‐lithiated aged samples than for the fresh materials cycled to the similar number of cycles. For example, for formatted S−LCO, the interphase resistance is 11.0 Ω while for S−LCO re‐lithiated after aging to SOH 70 % the same value is 23.2 Ω. The values for the formatted and aged D−LCO samples are 21.8 and 25.5 Ω, respectively. The result indicates that something occurs on the particle surfaces during the initial cycling that is not restored during the re‐lithiation of the recovered electrodes. In addition, the authors speculate that the increase in interphase resistance could possibly be caused by the degradation of the binder or the conductive carbon in the electrode. This would lead to decrease in conductivity within the electrode and therefore increase the active material/current collector interphase resistance in the electrode. The LCO particles could also lose their contact to the current collector due to multiple volume changes during the initial cycling. However, due to the mixed composition of the electrode, this is difficult to verify. The degradation of the electrode additives would nevertheless also explain the broadening of the peaks in the CV curves of the aged materials in Figure 5. The interphase resistance generally increases slightly upon cycling of the aged materials. This could be explained by side reactions occurring in the cell, for example, the formation of surface layers.

**Table 2:** Kinetic parameters of the investigated re‐lithiated LCOs.

Sample	*R*_S_ [Ω]	*R*_1_ [Ω]	*R*_2_ [Ω]
S−LCO form.	1.6	11.0	83.0
S−LCO 70 % re‐Li	4.2	23.2	240
S−LCO 10 c	1.7	21.8	715
S−LCO 70 % re‐10 c	4.6	20.5	7277
D−LCO form.	5.5	21.8	30.5
D−LCO 70 % re‐Li	5.0	25.5	43.2
D−LCO 50 c	3.6	28.3	109
D−LCO 70 % re‐50 c	5.6	32.3	177

The largest changes in the impedance data are observed in the mid‐frequency semicircles. When the re‐lithiated material is cycled, the semicircle increases, and this indicates that the charge‐transfer resistance of the electrochemical reaction increases. The increase in the semicircle size is much larger for S−LCO than it is for D−LCO, which indicates that the charge‐transfer resistance increases slower in the aged D−LCO. In our previous work,^[49]^ the Mg and Ti doping has been found to enhance the cycle life performance of LiCoO_2_ by decreasing the charge‐transfer resistance growth. Therefore, it is likely that the same process is behind the enhanced cyclability also after the re‐lithiation followed by the second cycling.

Interestingly, compared to the cycling of the fresh material the charge‐transfer resistance growth of the re‐lithiated S−LCO is huge already after 10 cycles, even when the resistance growth of the fresh material is larger for S−LCO than for D−LCO. On the other hand, while the increase in the charge‐transfer resistance is larger for the re‐lithiated than for the fresh D−LCO, the difference is not excessively large. This is explained by the XRD analysis. It shows that the bulk structure of the LCOs can be restored well, but after the re‐lithiation the stacking order decreases in both the materials, more in S−LCO. The poorer stacking order of the re‐lithiated S−LCO compared to the fresh S−LCO most likely leads to increase in the resistance, inducing the large growth after only a few cycles. This result shows that the properties of a material that enhance its performance in the initial cycling, greatly affect its properties during the second cycling as well. It also seems that the properties of the materials affect the second aging more strongly than the first aging.

As a summary, the electrochemical results show that the re‐lithiated D−LCO electrodes show promising rate capability and cyclability properties although the electrochemical performance of the re‐lithiated electrodes do not quite reach the performance of the fresh electrode. This would most likely restrict the use of the electrodes from the most demanding applications. However, the electrodes perform well and could be used under less demanding conditions, such as stationary energy storage for renewable energy production. Compared to the other reported LCO reuse methods, the slight difference between the initial and the recovered capacities of the LCOs is the largest disadvantage of this method. There are several studies in which the LCO is removed from the current collector, and in which the capacity is fully recovered.[^20^, ^25^, ^35^] However, the voltage ranges and C‐rates in these studies are usually lower than in our study, which could partially explain the difference. In addition, in our method no steps for separation processes are needed, which leads to reduced expenses and energy consumption. Material losses are also minimal due to the scarcity of the separation processes.

## Conclusion

In this work, the reuse of aged Mg−Ti doped and stoichiometric LiCoO_2_ (LCO) electrodes after electrochemical re‐lithiation was investigated. The materials were aged in LiCoO_2_/graphite pouch cells in the voltage range of 3.0–4.4 V and the properties of the aged electrodes were investigated. After this, the electrodes were lithiated in half‐cells and the performance of the cells was investigated. The post‐mortem analysis of the LCO electrodes shows that the capacity loss of the LCO materials is caused mostly by the loss of lithium due to solid electrolyte interphase layer formation on the graphite electrode. The crystal structure of the electrodes is restored upon re‐lithiation, except for the stacking order that is poorer for the stoichiometric S−LCO than for the Mg−Ti‐doped LCO. The re‐lithiated Mg−Ti‐doped LCO has an enhanced capacity retention compared to the re‐lithiated stoichiometric LCO. In the reusability tests, it is found out that the rate capability properties of the aged Mg−Ti doped LCO vary only a little from the rate capability properties of the fresh Mg−Ti doped LCO. In the cycling tests, the re‐lithiated, doped LCO has an enhanced capacity retention compared to the re‐lithiated undoped LCO and it is only slightly poorer compared to the fresh Mg−Ti doped LCO. The poor stacking order of the re‐lithiated S−LCO is concluded to explain the difference between the materials and the difference to the fresh electrodes. The possibility of degradation in the electrode additives is also discussed. It is concluded that an alternative recycling method for current Li‐ion battery electrode recycling methods could be the electrochemical re‐lithiation of a collected electrode. This would save resources as the LiCoO_2_ separation process from a binder, conductive carbon and Al current collector would not be needed. The selection of the recycled material is important, as a better performing aged material also performs better after electrochemical re‐lithiation. Mg−Ti doped LCO offers a promising option for this.

## Experimental Section

The aged pouch cells contained one negative‐positive electrode pair. Graphite (Hitachi) was used on the negative electrode and the investigated LiCoO_2_ (LCO) materials (Umicore Finland) on the positive electrode. The graphite slurry consisted of graphite, conductive carbon (Timcal Super C65) and polyvinylidene fluoride (PVDF; Kureha), in the weight ratio of 92 : 4 : 4, respectively, and it was coated on a copper foil with the loading of 6.0–7.2 mg cm^−2^. The LCO electrode consisted of LiCoO_2_, conductive carbon and PVDF in the ratio of 95 : 2 : 3, respectively, and was coated on an aluminum foil with the loading of 11.0–14.5 mg cm^−2^. *N*‐methyl‐2‐pyrrolidone (NMP; BASF, Life Science) was used as a solvent during the electrode preparation. The size of the graphite electrode was 65 mm×48 mm, the LCO electrode 61 mm×44 mm and the separator 68 mm×48 mm. 1 m LiPF_6_ in 25 : 70 : 5 ethylene carbonate (EC)/diethylene carbonate/propylene carbonate solution with 1 mol% vinylene carbonate and 1 mol% 1,3‐propane sultone doping (Golden Light Hi‐Tech Energy Storage Materials, JR‐02) was used as an electrolyte.

The aging of the pouch cells was done with a Neware battery cycler using the voltage range of 3.0–4.4 V and the C‐rate of 0.5 C. The cells were cycled until the desired SOH was achieved. After this, the pouch cells were dismantled in a glovebox with an argon atmosphere (*c* ${}_{O{}_{2}}$ <0.2 ppm, *c* ${}_{H{}_{2}O}$ <0.2 ppm). The electrodes were separated, rinsed three times in dimethyl carbonate (DMC) solution for 30 s each, dried and cut into Ø 14 mm spherical electrodes for characterization.

The composition of the LCOs was analyzed with a Thermo iCAP6500 inductively coupled plasma optical emission spectrometer (ICP‐OES). The crystal structures of the electrode samples were characterized with XRD (PANalytical X'Pert Pro MPD, CuK_α1_‐radiation). The vibration characteristics of the samples were analyzed via Raman spectrometer (Renishaw, inVia confocal raman microscope) using a 532 nm argon ion laser as the source of excitation in the range of 100–3200 cm^−1^. The particle sizes and morphology in the electrodes were investigated with SEM (Tescan Mira‐3, in‐beam secondary electrons, 5 kV). To ensure the conductivity of the samples, they were coated with 80 : 20 Au/Pd sputtering. A double‐aberration corrected JEOL JEM‐2200FS microscope equipped with a 200 kV field‐emission gun (FEG) and an in‐column energy filter (Omega filter) was used to perform the EELS measurements. A Varian AA240 AAS was used to measure the elemental composition of the graphite electrodes.

To perform the re‐lithiation and the electrochemical tests, the Ø 14 mm electrodes were assembled to half cells using Hohsen 2016 cases. 0.75 mm thick lithium metal foil from Alfa Aesar was used as a counter electrode, and 1 m lithium hexafluorophosphate (LiPF_6_) dissolved in 1 : 1 EC/DMC solution (BASF, LP30) as an electrolyte. A Whatman GF/A 0.26 mm thick glass fiber filter was used as a separator. After the assembly, the cells were left to stabilize for 24 h before the testing began. CV and galvanostatic measurements were done in two‐electrode cells and EIS measurements in three‐electrode cells.

CVs were measured using an Autolab potentiostat (PGSTAT302 N) with a GPES software. The voltage range of 3.0–4.5 V and the scan rate of 0.02 mV s^−1^ were used to measure three cycles per scan rate. In the re‐lithiation of the investigated electrodes, the C‐rate of 0.03 C was used to charge and discharge the cell once. The galvanostatic rate capability measurements were done using the Neware battery cycler. The rate capabilities of the assembled battery cells were measured in two voltage ranges, 3.0–4.3 and 3.0–4.5 V. The discharge C‐rates used in the program varied from 0.1 to 4.0 C, and the charge C‐rate was kept constant at 0.2 C. The C‐rates were calculated using the theoretical capacity of 160 mAh g^−1^. At least three parallel samples were measured to ensure the repeatability of the results.

EIS measurements were performed with an Autolab potentiostat (PGSTAT302 N) using an FRA software. The frequency range was 100 kHz–10 mHz and the alternating potential amplitude was 5 mV. The measurements were done at the open circuit voltage (OCV) corresponding to the SOC of 50 %, which was determined before the cycling.

## Conflict of interest

The authors declare no conflict of interest.

## Supporting information

As a service to our authors and readers, this journal provides supporting information supplied by the authors. Such materials are peer reviewed and may be re‐organized for online delivery, but are not copy‐edited or typeset. Technical support issues arising from supporting information (other than missing files) should be addressed to the authors.

Supplementary
