# Supplementary material for: Reuse of LiCoO2 Electrodes Collected from Spent Li‐Ion Batteries after Electrochemical Re‐Lithiation of the Electrode
Source: ChemSusChem. 2021 May 6;14(11):2434–44. doi: 10.1002/cssc.202100629 (PMC8252475; doi:10.1002/cssc.202100629)
Supplement: Supplementary file 1 — Supplementary [file CSSC-14-2434-s001.pdf]

ChemSusChem

Supporting Information

Reuse of $LiCoO_2$ Electrodes Collected from Spent Li-Ion Batteries after Electrochemical Re-Lithiation of the Electrode

Katja Lahtinen, Eeva-Leena Rautama, Hua Jiang, Samuli Räsänen, and Tanja Kallio*© 2021 The Authors. ChemSusChem published by Wiley-VCH GmbH. This is an open access article under the terms of the Creative Commons Attribution License, which permits use, distribution and reproduction in any medium, provided the original work is properly cited.

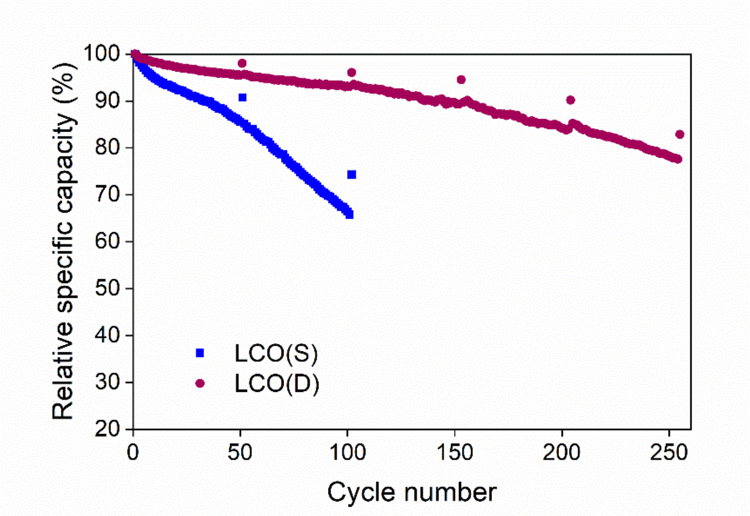

Fig. S1. The relative capacity loss of S-LCO and D-LCO in LCO/graphite pouch cells in the voltage range of 3.0–4.4V.

Table S1. Compositions of the investigated $LiCoO_2$s.

	S-LCO	D-LCO
Li/Co ratio	0.97	0.98
Product formula	$Li_{0.97}CoO_2$	$Li_{0.97}(Mg_{0.005}Ti_{0.002}Co_{0.994})O_2$

Table S2. Observed elements in the aged graphite electrodes.

**Graphite sample**	**Li (mg/g)**	**Co (mg/g)**	**Mg (mg/g)**	**Ti (mg/g)**	**P (mg/g)**
**S-LCO form.**	5.3	< 1	< 1	< 2	< 2
**S-LCO 90 %**	14.2	< 1	< 1	< 2	1.8
**S-LCO 70 %**	23.2	< 2	< 2	< 4	9.4
**D-LCO form.**	5.2	< 1	< 1	< 2	< 2
**D-LCO 90 %**	9.5	< 1	< 1	< 2	1.4
**D-LCO 70 %**	18.2	1.9	< 1	< 2	12.0
